# Sustainable Solutions for Advanced Energy Management System of Campus Microgrids: Model Opportunities and Future Challenges

**DOI:** 10.3390/s22062345

**Published:** 2022-03-18

**Authors:** Hafiz Abdul Muqeet, Haseeb Javed, Muhammad Naveed Akhter, Muhammad Shahzad, Hafiz Mudassir Munir, Muhammad Usama Nadeem, Syed Sabir Hussain Bukhari, Mikulas Huba

**Affiliations:** 1 Department of Electrical Engineering, Punjab Tianjin University of Technology Lahore, Punjab 54770, Pakistan; abdul.muqeet@ptut.edu.pk; 2 Department of Electrical Engineering, Muhammad Nawaz Sharif University of Engineering and Technology, Multan 60000, Pakistan; haseebjaved1996@yahoo.com (H.J.); dr.shehzad@mnsuet.edu.pk (M.S.); musamanadeem1@gmail.com (M.U.N.); 3 Department of Electrical Engineering, University of Engineering and Technology, Punjab, Lahore 58890, Pakistan; naveed.akhtar@uet.edu.pk; 4 Department of Electrical Engineering, Sukkur IBA University, Sukkur 65200, Pakistan; mudassir.munir@iba-suk.edu.pk (H.M.M.); sabir@iba-suk.edu.pk (S.S.H.B.); 5 Institute of Automotive Mechatronics, Faculty of Electrical Engineering, and Information Technology, Slovak University of Technology in Bratislava, 812 19 Bratislava, Slovakia

**Keywords:** smart grid, energy storage system, campus microgrid, distributed generation, distributed energy resources, demand-side management

## Abstract

Distributed generation connected with AC, DC, or hybrid loads and energy storage systems is known as a microgrid. Campus microgrids are an important load type. A university campus microgrids, usually, contains distributed generation resources, energy storage, and electric vehicles. The main aim of the microgrid is to provide sustainable, economical energy, and a reliable system. The advanced energy management system (AEMS) provides a smooth energy flow to the microgrid. Over the last few years, many studies were carried out to review various aspects such as energy sustainability, demand response strategies, control systems, energy management systems with different types of optimization techniques that are used to optimize the microgrid system. In this paper, a comprehensive review of the energy management system of campus microgrids is presented. In this survey, the existing literature review of different objective functions, renewable energy resources and solution tools are also reviewed. Furthermore, the research directions and related issues to be considered in future microgrid scheduling studies are also presented.

## 1. Introduction

Distributed generations (DGs) have the potential to overcome the problems of energy systems all over the world, such as power stability, system reliability, network overloading, greenhouse gas emissions, and high consumption cost. The energy management system of large commercial building microgrids has created problems to minimize the network load deviation and operational cost [1]. The energy management system (EMS) of the multi-energy microgrid (MG) can reduce the operational cost and is able to enhance energy utilization efficiency [2]. However, the distribution generations (DG) consist of renewable energy resources (RER) such as biomass, photovoltaic (PV), wind turbines (WT), fuel cells (FC) accompanied by non-renewable energy sources such as diesel generators (DiG), gas engines (GE), micro-turbines (MT) [3]. 

**Figure 1:**
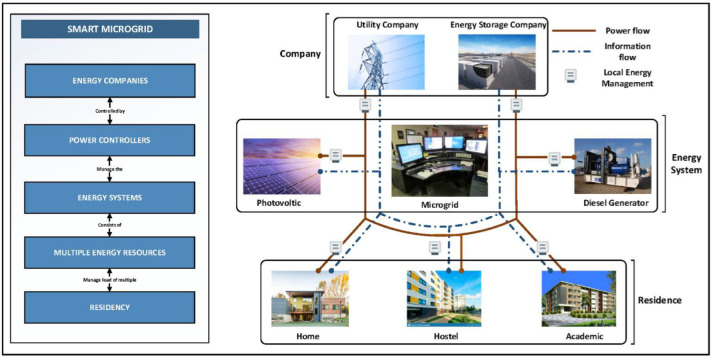
Generic Microgrid model.

Microgrids have different types of systems, such as flexible load, DGs, and energy storage systems (ESS). The generic microgrid model is described as the model as illustrated in Figure 1 that contains Solar PV, Diesel generator, grid, and energy storage company [4]. It also contains controllers that efficiently deal with the system by controlling the load to increase the solar output. This model is a bi-directional power flow as it takes the load from the homes, hostels, and academic departments [5]. 

In this model, those users who act as consumers and prosumers will be dealt with an intelligent energy management system. It is a generally understood that a microgrid that takes load from the user efficiently is a better maintained, reliable, and efficient microgrid system. One of the general microgrid models is also shown as an example in Figure 1.

The DG depends on the control of the distributed energy resources (DER) and the optimal scheduling of the microgrid. The optimal scheduling of power generation expressively affects the stability of the energy system [6]. Different scheduling techniques of the power system are used to improve the power quality and voltage control of microgrids based on the real microgrid solution with multiple implementation scenarios that aimed to get green energy and to make an efficient smart campus to achieve sustainable energy for the campus microgrid with the reduction in GHG emissions [7]. 

Microgrids face different types of problems due to the variation in demand side and fluctuations in voltages and frequencies. Energy management systems (EMS) normally face microgrid problems by the insufficiency of energy production sources. It aims to define the optimal usage of DG to feed the electrical loads [8]. EMS operates in centralized and decentralized modes. Centralize modes are those in which the power exchange of microgrids mainly bases on the price of markets. The decentralized mode is opposite from the centralized mode because of autonomy power exchange without the market price limitation [9]. Stability, efficiency, and energy protection are also the critical issues of microgrids due to reverse flow of power of generation units, voltage fluctuations, microgrid transient modes, drastic frequency variations in islanded operating mode, and supply-demand microgrid uncertainties in which high levels of angle droop are required for proper load sharing, especially under weak system conditions. EMS also contains multiple challenges. To overcome these challenges, a detailed overview of some microgrids has been developed to discuss major issues in the energy management systems [10]. 

A general overview of some microgrids with their components installed are given in Table 1 to give us a summarized analysis of various microgrids with the comprehensive review considering load type, optimization techniques, and results:

The main contributions of the survey paper are:

1. This paper focuses on the survey of optimal scheduling of the distributed energy resources with the various campus microgrids;
2. It also presents the scheduling of different energy resources with a comprehensive review of the energy management of various campus microgrids at different locations;
3. EMS of microgrid has been reviewed considering the distributed generation, renewable energy resources, demand-side management (DSM), and ESS;
4. Energy management and optimal scheduling of microgrids have been evaluated concerning objective functions (OFs), optimization techniques, simulation tools, and constraints. A comprehensive research challenges and issues are discussed.

This study also aims to critically analyze many microgrids to give an overview of multiple campus microgrids, to analyze their campus energy management systems, and provide some solutions for them to optimize their campus. It focuses on the field of campus microgrids with an emphasis on industrial microgrids and prosumer microgrids. Nowadays, many power producers are aimed at producing their power energy supply often termed as “Prosumers”. The contribution of this novel research is to help other researchers in the field of the energy management of campus microgrid as it briefly describes the systematic overview of various literature papers with the consideration of their installed system and the approaches with the focus of multiple solutions are presented here. This novelty also helps in exploring a new dimension of distributed generations. The innovative approach of this paper is that it is also helpful for those researchers who aim to deliver some novelty in the field of campus microgrids, demand-side management, and optimal scheduling of distributed microgrids.

This paper presented the literature review of distributed generation (DG) which has been classified into five categories:

(1) Solar PV;
(2) Wind turbine;
(3) Fuel cell;
(4) Diesel generator;
(5) Energy Storage System.

This survey paper is further arranged as described: in Section 2, the energy management of campus microgrids with distributed generation. The optimal scheduling of microgrids is presented in Section 3. The simulation tools for optimal scheduling of microgrids are reviewed in Section 4. Lastly, research challenges and the conclusion are presented in Section 5 and Section 6.

## 2. Energy Management of Campus Microgrids with Distributed Generations

A microgrid mostly consists of an energy storage system (ESS), distributed generation (DG) resources, and load. Distributed generation has various types of technology for the generation of electricity, such as combine systems, solar panels [31]. To analyze the energy management of microgrids, we can discuss the self-resilience of microgrids as it makes the microgrids self-reliant [32]. In the centralized system, self-reliance provides communities with an efficient way to deal with the independent energy suppliers with the usage of fossil fuels. It provides remote community members an easy way to connect with the utility and to access the electricity more appropriately. Self-reliance helps the microgrid function as a self-reliant power producer [33].

**Figure 2:**
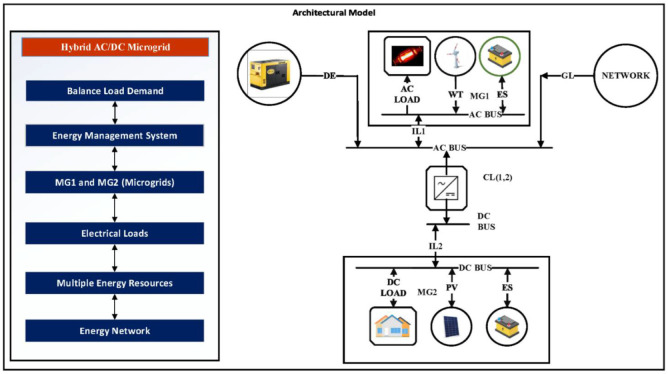
Architectural Model of an EMS Hybrid AC/DC Microgrid.

On the other hand, a combined system consists of WT, DiG, FC, and PV is developed in Figure 2 to show the self-resilience of microgrids and how they manage the AC or DC load in the communities.

In Figure 2, Hybrid AC/DC microgrid units are connected to each other to balance the demand loads with the help of EMS. In MG1, battery, wind, and loads are connected with AC-BUS. Similarly, the components of the MG2 are connected with AC BUS (1–2), while CL (1–2) is the converter that is connected with the system. This model represented the microgrid systems connected with one another that aim to manage the load of the communities independently. 

Now, we will discuss the microgrid systems with multiple solutions which have been presented for different EMS systems, optimization techniques, and various renewable energy resources. Several authors have reviewed these distributed generations for different microgrid systems that are briefly described here: 

Shahidehpour et al. [34] devised the energy management model to reduce the operation cost of the microgrid. For this purpose, the high-reliability distribution system technique was implemented in this Illinois campus (IIT). On the campus, the microgrid has distributed generation (DG), distributed energy resources (DER), controllable load, and energy storage systems (ESS). The proposed system was comprised of distributed generation. MG contains different HRDS switch for the reliability indices. Using this technique, the annual operation cost of campus microgrids reduce from 140,497 $/year to 119,236 $/year because the purchasing cost of energy fluctuates every hour. From this technique, it cannot focus on other parameters like uncontrollable loads, smart loads, and multiple energy storage systems at once. An effective solution with an improved distribution technique like soft computing techniques, fuzzy modeling techniques, or load flow techniques must be developed and implemented to further reduce the operational cost of the campus microgrid. 

The prosumer campus microgrid is presented by Muqeet in [3] to financially save the consumer’s operational cost with energy storage system (ESS) and distributed energy resource (DES). Three scenarios are present in this paper for the consumer:

1. With only a grid attached;
2. With photovoltaic (PV) source and ESS along with the grid source;
3. With Wind energy, PV, and ESS along with the grid source.

**Figure 3:**
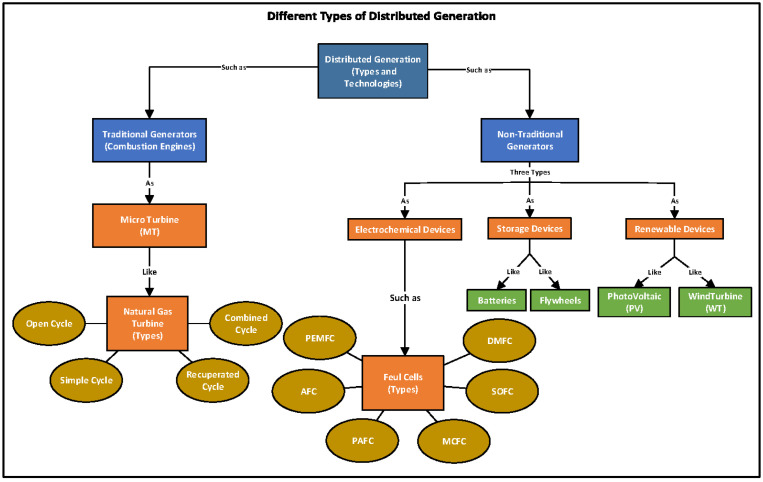
Architectural Model of an EMS Hybrid AC/DC Microgrid.

MILP technique simulates the optimal schedule for the power system in MATLAB. After the energy management, the system’s operational cost reduces 67.91% per day by integrating the Wind, PV, ESS, and grid energy. However, it lacks additional renewable energy resources which can be incorporated with the system such as Hydal and it can also be simulated with more advanced techniques like neural networks, deep learning, or any advanced technique. Various types of distribution generation is illustrated in Figure 3 in which distributed generation [35] consists of two types of traditional and non-traditional generators which are also subdivided into further categories in which electrochemical devices such as fuel cells consist of polymer electrolyte membrane fuel cells (PEMFC), direct methanol fuel cells (DMFC), alkaline fuel cells (AFC), phosphoric acid fuel cells (PAFC), molten carbonate fuel cells (MCFC), solid oxide fuel cells (SOFC), and reversible fuel cells (RFC). 

### 2.1. Solar PV in Campus Microgrids

PV systems are used to generate electrical energy with the help of solar energy. The PV system consists of more than one PV panel, electrical and mechanical connectors to produce an electrical output. Panels are connected to produce the required amount of current and voltage [14]. 

Some authors have also reviewed PV systems of different campus microgrids and various energy systems.

Reyasudin et al. [12] devised the EMS (Energy Management System) model for the University of Kuala Lumpur, British Malaysian microgrid, which aims to reduce the operational cost of the microgrid. Energy storage systems (ESS) and Photovoltaic (PV) are used in the microgrid to meet the campus load demand. The HOMER software was used here to evaluate and analyze the environmental, economic, and electrical performance of the Hybrid Renewable Energy System (HRES). However, it can also be simulated with more advanced software like PVsyst [36], PVsol, or PV modeling software to achieve more accurate results.

Another energy management system is presented by Leskarac in [14] for the huge commercial building microgrid to reduce the network load variation and operational cost. It is proposed by the bi-level linear model that contains mobile storage (electric vehicle), stationary storage, microturbine, fuel cell, solar PV, and solved using the flower pollination algorithm (FPA) in MATLAB. The simulation results of the grid-connected mode and the isolated mode of the microgrid was studied and improved. However, the author does not address the frequency regulation or the power quality. It can also be solved with more advanced optimization techniques like Spiral optimization (SPO) 2013, Artificial swarm intelligence 2014, Golden Eagle Optimizer (GEO) 2020, and Jellyfish Search (JS) 2021, etc. [37].

An optimal system is introduced by Kumar in [38] on the (Nanyang Technological University (NTU), Singapore) campus microgrid (MG) includes photovoltaic (PV), natural gas micro-turbine (MT), Electric vehicles (EVs), and a fuel cell (FC). Here, the author discusses how to manage the system’s energy and elaborates on how to achieve the demand response (DR). They also describe how to achieve the output level of solar PV using the NTU campus’ vehicle-to-grid technology using a PV system. On a typical day, the building serving transformer support an average of 17.3 kW of additional EV loads. Approximately MG 33% significantly supports the campus and EVs loads. However, it can also be addressed with the incorporation of wind and hydel resources, if possible. The author did not focus on the specific demand response programs like Incentive-based programs [39], Real-time pricing [40], Market-based programs [41], Price elasticity [42], and Price-based programs, etc.

Another system is devised by Esmaeili in [43] that enhances the optimal scheduling of multi-microgrids (MGs) in which the distribution system is enhanced by energy storage systems (ESS) and demand response (DR) programs. The microgrid and Distribution System Operators are the core objective discussed here because the upper level reduces the operational cost from DSO and the lower level increases the profit of MG with the help of energy management (EM). Mixed-Integer Second-Order Cone Programming (MISOCP) is formulated as an optimization problem which is conducted by the General Algebraic Modeling System (GAMS) language and resolved by the CPLEX solver. Market prices are relatively moving upward, so MG owners choose to install their distributed energy resources first, which includes microturbine (MT), Photovoltaic (PV), and responsive load, and then transfer the power with the others connected DSO and MGs. However, it focuses only on the market-based price demand response, and it can also consider other demand response programs like incentive-based programs or real-time pricing (RTP) schemes. Moreover, MISOCP can also be implemented on other modeling tools like AIMMS, AMPL, Mathematica [44] or APMonitor, etc. to get better results. 

### 2.2. Wind Turbine in Campus Microgrids

Wind turbines (WT) generate electrical energy by wind power. Wind turbines are constantly dependent on airflow and their output vary according to the speed of air. Some authors have also reviewed wind systems on different campuses and islanded microgrids:

Liu et al. [45] presented the ESS sizing technique with a comprehensive consideration of DGs, loads, and energy storage. DGs include wind turbines, Solar PV panels, electric vehicles, and combined heat and power (CHP) generation. A two-layered hybrid ESS (i.e., lead-acid battery). As shown in Table 2, several scholars have employed these optimization techniques to obtain the best solutions.

Li-ion battery Supercapacitor with three types of storage is built according to their power density, load classification, and Demand Response (DR), which is the main tool for attaining greater operational efficiency, reducing capital, and operational costs in MG resource size optimization. It uses different types of loads which are suitable for different kinds of energy storage systems that are hybrid and aim to improve energy storage systems’ economy and reliability. 

Moreover, huge differences in load variation during different periods are provided by many types of storage, while Lithium-ion batteries take priority over lead-acid batteries. This method reduces battery replacement time during the timespan of the MG. When the EV and DSM plan are taken into account, the load curve is smoothed, which results in a significant amount of profit, including the efficiency of the system. However, it lacks battery degradation cost with an economic analysis to predict the battery degradation according to time and it also focuses on a two-layered hybrid ESS system which also lacks the selection of some advanced energy storage systems such as Siemens Junelight Smart Battery SB–(3,3), Battery flex AC-1 1.3 (6.0 kW, 4.8 kWh) [55], or REACT2-5.0–12 kWh–AC or DC, etc. 

On the other hand, MG performance was observed by Baron in [56], where the research aimed to increase the optimal scheduling of various types of grids. It included operational costs of the system and costs associated with the loss of energy storage. The author suggested this to avoid all the renewable energy transmission costs and cost of storage systems. It is noteworthy that this pattern has been observed in wind and solar energy production systems. Thus, this research provides the project operator with a tool to determine the best operational phase of the MG by considering various events of the batteries’ useful life. However, it does not focus on providing the optimal battery size which may increase the operational cost of the microgrid. Thus, to reduce the operational cost and other costs, a sizing approach is focused on various renewable energy resources. To calculate optimal sizing approach for systems, various advanced optimization tools are available which can be used in this regard such as PVsyst, PVsol, or HOMER pro [57,58]. 

Another optimal scheduling model is proposed by Du in [59] that optimally schedules and operates the microgrid clusters of multi-microgrids’ energy and establishes an optimal scheduling system to reduce the system operating costs for the microgrid (µG). The µG includes wind turbines (WT), combined heat and power (CHP), electric refrigeration (EC), photovoltaic (PV), electric boilers (EB), and other equipment. It is solved by the CPLEX solver for model optimization solutions under the GAMS platform. The total daily operation cost is calculated for case 1 is $29,033.6378 and for case 2 is $29,415.1206. Both the cases are analyzed to select the optimal system. However, the system can also be solved by a Gurobi solver to get better results and many other optimized renewable energy resources must be incorporated such as wind or hydro to further reduce the operational cost of the microgrids. 

Now, Huang presented the microgrid configuration in [60] and introduce a power consumption schedule optimization by a Stackel-berg game which models the 2- rational decision-maker that relates among each other for the microgrid and can manage the energy consumption scheduling problem. It makes the decision for the microgrid, as the supreme leader, which leads to an advanced optimization problem to maximize the installed number of micro-turbines, photovoltaic (PV) units, wind turbines, and batteries. Microgrid configurations in residential buildings are used to validate the efficiency of two-level scheduling and two-level classified algorithms [61]. 

By comparing four two-tier algorithms, the experimental results show that the Stackelberg game model optimizes the timing of smart home and microgrid configuration simultaneously. Results show that the simultaneous optimization of power consumption and optimal scheduling of the microgrid configuration can significantly optimize the cost of configuration, even when there is little support for the public network [62]. Furthermore, the simulation results indicated that the proposed model is suitable for customer engagement to reduce consumption, such as changes in usage time and energy levels. However, the microgrid configuration can also be improved by implementing both Stackelberg and Cournot models together [63]. As the microgrid decision support system needs to be improved because it is the central brain of the system that controls everything [64]. 

### 2.3. Fuel Cell (FC) in Campus Microgrids

Fuel cells work like batteries, but they do not need recharging every time. It is an electrochemical cell that produces electricity from chemical energy. Most commonly used fuel cells are the PEMFCs (Proton exchange membrane fuel cells) which is common nowadays because it operates at very low temperature (−20 C) to (1000 C) and it can operate quickly in ideal condition to full load conditions [65]. 

Some authors have also reviewed fuel cells that are installed at different campus microgrids and various locations:

Bouakkaz et al. [66] proposed an energy management approach that optimally improves batteries’ lifetime by optimizing energy consumption at home with a unique fuel system connected to a fuel storage system consists of (photovoltaic, batteries, diesel generator, and wind turbine). Recently, optimization algorithms have attracted lots of attention to solve various engineering problems and some of them have high accuracy and lead to higher efficiency and promising results. The rain flow algorithm is used to compute the number of life cycles of the battery, but the problem is solved by the optimization technique called the PSO (Particle Swarm Optimization) algorithm. This optimization minimizes the number of battery cycles throughout the whole day by maintaining the charging/discharge process that aims to increase the battery’s life cycle. The simulation results are obtained to show the efficiency of the proposed management approach to optimize the battery life cycle to more than 38%. However, the system lacks optimal sizing of batteries or battery degradation cost which also affects improving the battery life and reducing the cost of the energy storage systems. It can also incorporate more advanced techniques like Artificial bee colony algorithms, multi-swarm optimization, or Swarm intelligence, etc. [67].

The distributed energy storage system (DESS) is addressed by Kim in [68] to propose a low-cost planning method for the microgrid group. The proposed planning algorithm operates the community microgrids, which consists of large ESS & large-Scale Fuel Cells (LFC) that make the planning procedure while considering the variability of net load and CDESS market procedure is operated for the DESS system. In the LFC and LESS planning problem, the net load variation is formulated as a function of the amount of electricity exchanged with the external electrical grid. In the case of the Customer DESS market operation scheme, the market scheme is depending upon the price-signal market. The simulation results show that the LESS operation cost is reduced to operate the community microgrids. However, it can also focus on the expansion planning of the active distribution network while using enhanced heuristic optimization techniques. More, the system does not focus on the economic analysis and it can also focus on new market schemes to further reduce the operation cost of DESS. 

A non-linear model is proposed by Mohsin [69] to optimize the energy management of emission-free ships (EF-Ships) with hybrid CI/ESS/FC as storage energy resources, focusing on the decaying life-span of fuel cells (FC), fuel systems, and energy storage (ESS). Aging factors and total operational costs of FC and ESS are analyzed. This article presents an energy management scheme for EF-Ships with combined FC and ESS as power resources. The proposed method deliberates both the aging factors of the FC and ESS and the ship’s operation cost, and the problem attempts to find the optimal solution for the energy planning program that reduces the operating costs while taking into account the limitations of aging and decaying of the equipment [70]. The suggested SMPC method’s efficiency in processing rapid ups and downs in weather forecasting and the GAMS software tries to solve the suggested optimization problem calculated during the simulation process. The obtained simulation results indicate that the effectiveness of the recommended model to comply with the FC and ESS decaying/aging limitations while minimizing the operating costs of the system by 4.67%. However, it does not focus on the degradation cost of the energy storage systems and their optimal sizing approach. More, other tools are also available which can give better results than GAMS for modeling such as AIMMS, AMPL, APMonitor, or Mathematica, etc. [71]. 

However, a comprehensive EMS (energy management system) model is devised by Violante in [72] for a separate micro-grid that incorporates thermal energy resources, such as thermal storage systems (TSS), combined heat and power (CHP) units, heat pumps, boilers, and heat (HP), taking into account the thermal load model, is recommended in this article. The advanced SMEs are verified and tested with an actual test bench micro-grid situated in Italy and Bari, which provides both the heat and electricity in a building located in Politecnico de Bari. The recommended EMS is intended to reduce the fuel costs of the microgrid system, and it models properly for cogeneration units. This model is optimized by the optimization problem called the (MILP) technique that is easily manipulated with viable solvers, making the EMS system suitable for online applications. MILP is an important technique in optimization methods utilized in various applications [73]. The simulations are performed for altered winter days that also have demonstrated the cost-effective benefits. Models of thermal systems in a micro-grid EMS, resultant in the profitability of the daily fuel costs. This significantly increased the total cost by more than 40% compared to the suggested EMS. Consequently, the incorporation of thermal systems into this micro-grid EMS has proved to be valuable. Moreover, it lacks the utilization of modern techniques like deep learning or artificial neural network, and it can also incorporate other thermal energy resources, if possible, like geothermal energy resources which give beneficial results. 

Now, various number of fuel cell (FC) operated cars are reviewed by Alavi in [74] that can be seen as an energy production that is distributed within an islanded microgrid, and proper fuel cell power planning can keep up the power stability of the MG. The MM and DF MM methods are able to generate the FC incorporated power by reducing the operation cost of the system. Simulation results show that microgrids consider network topology with low-level control models, develop the distributed control architectures for the microgrids in grid-connected modes, and also considers the assembling of fuel cell vehicles using the ADMM technique. However, it can also be modeled by sequential quadratic programming, sequential linear programming, and sequential linear-quadratic programming and can also be simulated in Accord.NET (C# augmented Lagrangian optimizer), or ALGLIB (C# and C++ preconditioned implementations of augmented Lagrangian solver), etc. [75]. 

### 2.4. Diesel Generator in Campus Microgrids

Diesel generators convert the chemical energy to mechanical energy that contains diesel fuel, through combustion. The mechanical energy in the generator rotates the crank that can generate electricity. Electric charges are made in the electric wire by moving in a magnetic field, this is how a diesel generator works. Here, the Diesel generator (DG) is characterized based on efficiency and fuel consumption. 

Some authors have also reviewed diesel generators that are installed at different campus microgrids and various locations:

Rural areas of most developing countries are disconnected from electrical energy but not at all times, because without electrical power, it would not be possible to survive [76]. Therefore, Arthur introduces a more realistic model for the rural area appliances and the energy management optimized for the microgrid. Renewable energy resources, such as diesel generators and energy storage systems (ESS), fully support running a microgrid. However, the results are simulated in MATLAB software using the Linear Programming technique to maintain the load’s demand response (DR). HOMER software can also calculate the fuel consumption of the running generator on an hourly basis that is also formulated in [77]. However, more advanced techniques must be utilized like MILP [78] or Deep learning [79], etc. Homer pro can also be utilized in this regard to effectively manage the microgrid [80]. 

On the other hand, an EMS (Energy Management System) model is developed by Krishnan in [21] for the industry microgrid (MG) to fulfill the industry’s appliances’ peak time that consumes power. Here, MG includes renewable energy sources (RES), diesel generators (DG), interruptible loads (IL), battery energy storage systems (BEES), and flexible loads (FL). The MILP (Mixed-integer linear programming) technique is used to simulate the energy management of industry load in MATLAB. Results show that optimal scheduling of the pump is improved, and system cost is reduced significantly while considering economic savings. However, smart loads, controllable or uncontrollable loads are not addressed here, and they must be addressed. Additionally, modern optimization methods can be utilized like the Flower pollination algorithm [81] or Harris Hawk’s optimization [54] rather than MILP to further optimize the system.

However, an effective operative model for a utility grid is presented by Karimi in [82] that is attached with the microgrid considering different energy generation resources consists of Diesel Generator (DG), Energy storage system (ESS), Wind Turbine (WT), Photovoltaic (PV), and Demand Response (DR) which is implemented by a mixed-integer linear program (MILP) technique. The GAMS technique was also used to resolve the multi-tasking optimization problem for energy management. However, the author does not focus on the optimal power flow or optimal energy exchange among grids. Power quality and voltage regulation [83] must be focused on here to get a more effective approach for the given system.

Another power system is focused in [84] in which the BESS system is integrated into the MG to ensure a more sustainable and economical system. The operational cost of the remote microgrid is minimized by cost-effective planning during consideration of the optimal battery size. Although fast discharging results in battery life decaying: as further energy sources are expected to use the battery size with optimal lifetime and energy storage, economic consideration in the isolated microgrid must be considered to deliver reliable service to the customers. The present study solved the economic planning problem between battery storage and diesel generators, considering battery degradation cost in real-time, ensuring reliable service. However, the selection of BESS must be addressed to find an optimal battery energy system for the MG to further reduce the energy cost for the system. The author mentions the optimal sizing approach for the BESS system but it lacks focus on high energy consumption usage from PV as it is the vital source to reduce the electricity cost for the microgrid [85].

Now, a smart charging program is proposed by Fouladi in [86] for the PHEVs (plug-in hybrid electric vehicles) to reduce GHG emissions of the utility grid, and it also reduces the high power consumption from the main grid by the increased usage of the RER/DER. Diesel generators, batteries, photovoltaic (PV) arrays and wind turbine (WT) are attached with the microgrid and properly integrated with the remaining grid, considering the system’s overall operational constraints. The suggested power management scheme allows V2G (Vehicle-to-Grid) and G2V (Grid-to-Vehicle) operating systems to be used by the MG Aggregator PHEV for support services. Consequently, the effects of the V2G operation mode and G2V operation mode of PHEV (WEG) on microgrids are examined. The simulation results show that the V2G operation mode and G2V operation mode of the EV charging stations are studied thoroughly, which enables it to run as an efficient source for the EV. In this paper, two scenarios are planned to assess the suggested power management’s efficiency and compare their results with those of the previously reported method. The proposed power management technique has proven to allow charging of PHEV depend upon the maximum integration of RER and DER; therefore, it reduces the power released from the utility grid even though the PHEV entry level is high. However, it does not focus on the price-regulated electric vehicle charging or discharging strategy for the V2G and G2V operation modes [87], and this must be addressed.

### 2.5. Energy Storage System in Campus Microgrids

An energy storage system is defined as the energy produced for later use that aims to reduce power energy imbalances between demand and power production. A device that stores electrical energy that is generated by any generator is generally termed a battery [88]. The microgrid that contains storage systems also contributes to the energy management of microgrids that provide the necessary information and efficient control system with essential functionality, which guarantees that both the generation side and distribution systems provide the electrical energy at nominal operational costs [89]. 

Some authors have also reviewed energy storage systems that are installed at different campus microgrids and various locations:

Stina et al. [90] presented an energy storage solution for the Tezpur University based in NE (North-East) India. This study consists of a DSM (Demand Side Management) system, an EMS (Energy Management System), and an ESS (Energy Storage system) with the integration of a Bio-mass power plant with a co-generating gas engine. The proposed system analyzed the cost minimization by reducing the usage of diesel engines and maximize the usage of PV-plant (1 MW) that was installed at the campus. Data were gathered to determine the economic analysis of the system so that profitability could be determined. By evaluating the data, an assessment has been developed that by a proper EMS, and an efficient ESS reduces the cost of electricity annually for the Tezpur Campus. Results revealed that the reasonable size for the lithium-ion batteries of BESS is 127 kWh at substation 4 and 90 kWh for the substation E4T microgrid. By this proposition, it is determined that it manages the campus load effectively and reduces the cost yearly. However, the proposed system lacks an optimal sizing of BESS which is an essential element in the energy storage system. To increase profitability, an effective sizing approach needs to be adopted and an applicable approach is needed to increase the high consumption of renewable energy resources [34]. 

In [91], the university installed a smart grid project at the MONASH campus, North Carolina, US. It consisted of 1 MW of Solar PV, 1 MWh of the energy storage system, and EV charging stations for 20 buildings. The main objective was to manage the bills of customers and to monitor the energy in real-time scenarios. However, Chongxin [92] overcomes the problems of a microgrid with multiple DER’s by optimally applying day-ahead scheduling of active/reactive powers. It included EV, energy storage systems, wind systems, PV, gas turbines, and loads for the Nanjing University Microgrid. The author analyzed it with the TOU (Time-of-Use) price approach. Load and renewable resources were predicted and modeled with an Deep Q-Learning-based optimization technique. It decided on the interval variable that sets the active/reactive power for the system to mitigate fluctuations. It finally resulted in the optimal schedule of the microgrid with multiple DER. Both authors have tried their best to install a smart grid project for the campus but they did not focus on the power quality [93] or voltage regulation [94] for the campus microgrid. An effective decision support system must be adopted that effectively manages the power flow among grids and a real-time pricing technique must be implemented.

Finally, Binod Koirala highlights key factors in [95] to improve the ICES (Integrated Community Energy Systems) with the consideration of power grid access, supportive incentives, voltage regulation, and structural design improvements. In this paper, several techno-economic perspectives are considered such as optimal energy storage devices, ancillary services, sustainability and flexibility, and cost-benefit analysis. Finally, it described the feasibility analysis of ICES technologies and the benefits of ICES in energy trends. However, the author did not focus on the optimal sizing parameters for the energy storage devices [96]. If such parameters are focused, it will improve the battery lifetime.

## 3. Microgrid EMS Objective Functions and Constraints

### 3.1. Objective Functions

EMS model manages various objective functions of the microgrid is described in Table 3. Start-up, shut-down, fuel, and maintenance costs are the microgrid operational costs [34]. It will help in analyzing multiple objectives for the campus microgrids while focusing on the objective functions table whose main objective is to describe the main components of various literatures paper that calculates the operational cost, net present cost, or any type of generation cost for the system. Its advantages or disadvantage can be analyzed in such a way that some objective functions minimize the energy generation cost or maximizes the utility functions while some respond vice versa.

### 3.2. Constraints

In Table 3, each optimization considers its own constrains, but there are two types of constraints in a constrained optimization problem which are important to be mentioned, such as OPF: equality and inequality. Equality constraints must be followed at all times. They are always “binding,” in other words. The real and reactive power balancing equations at load bus, for example, must always be met (at least to a user-defined tolerance) in the OPF, as must the area region MW interchange limitations [108]. Inequality restrictions, on the other hand, may or may not be enforceable. A line MVA flow, for example, might not have been at its full capacity, and a generator’s actual power output also may not be at its max capability. Multiple constraints create difficulties in the working of a microgrid. EMS help to balance the system if constraint does not affect the system. Constraints lead to damage to every part of the system [109]. Start-up of the system, charging, discharging of the energy storage system, shut-down of the system, feeder currents, the voltage at buses, frequency security aspects, reserve constraints, and ramping limits are also constraints.

### 3.3. Uncertainty Parameters

Different parameters are involved that reduces the power energy exchange between the microgrid and the main grid. The system involves multiple parameters that reduce the performance of the MG and to reduce the energy exchange, an IPI (Independence Performance Index) needs to be calculated. It affects the reduction in multiple factors and parameters like system losses, greenhouse gas emissions, and voltage drop in the system [110]. 

However, MG targeting in the reduction in the daily operational cost, maintenance cost, and miscellaneous cost of the system. The system contributes to the programs like DR (Demand Response) that manage the flexible or non-flexible loads effectively [111]. Net metering is also carefully undertaken to exchange the power among the utility while lessening the cost of non-supply of energy to the consumer end with the consideration of DER investments [112]. A robust optimization method is implemented that considers many errors that forecast in the future for consideration of load, market prices, and variable renewable generation [113]. 

## 4. Multiple Approaches Used for Optimal Scheduling of Campus Microgrids

### 4.1. Heuristic Approaches

The heuristic approaches are quick decision-making techniques used to resolve the optimization problem of the systems [49]. Some of the methods are meta-heuristics that have a different way of exploration and exploitation [114]. The bi-level linear model contains mobile storage (electric vehicle), stationary storage, microturbine, fuel cell, solar PV and is solved using the flower pollination algorithm (FPA) in MATLAB [115]. Similarly, adapted genetic algorithm according to the multicellular organism mechanism (GAMOM) used for the operation of the microgrid. Results are based on the applied method because every technique is more reliable than another such as particle swarm optimization (PSO) [116], genetic algorithm (GA) [117], and Teaching learning-based optimization (TLBO) [118]. Mixed-integer linear programming (MILP) is also used here to simulate the industry load’s energy management [119]. The MILP method obtains and solves the start-stop status, operational cost of each microgrid unit, and ESS, and then compares it [120,121].

### 4.2. Multiagent System (MAS)

A computerized system comprised of several interacting intelligent agents is known as a multi-agent system (MAS or “self-organized system”). Multi-agent systems can tackle issues that a solitary agent or a monolithic system would find difficult or impossible to address. Methodic, functional, procedural methods, algorithmic search, and reinforcement learning are all examples of this intelligence. Let us take an example presented in one literature: Load distribution control in an inverter-based MG using a completely distributed MAS-based approach with limited communication. Detection algorithm that requires less data transfer than most existing MAS-based load distribution studies to detect global microgrid information [122]. The rain flow counting algorithm is used to determine the number of cycles of the battery, but the optimization problem is solved using the particle mass optimization (PSO) algorithm [123]. MAS deals as a self-organized system that is very intelligent controlling multiple agents at a single time.

### 4.3. Mathematical Methods

#### 4.3.1. CPLEX Solver

CPLEX Optimizer offers flexible and high-functioning mathematical programming solvers for linear programming, quadratic programming, and mixed-integer programming, etc. Various mathematical programming is solved by a CPLEX solver like Mixed-Integer Second-Order Cone Programming (MISOCP) optimization problem is formulated using the General Algebraic Modeling System (GAMS) language and solved by the CPLEX [124]. Mixed-integer linear programming problems are linearized and formulate the electrical flow and natural gas equation using the General Algebraic Modeling System (GAMS) algorithm techniques [125]. Demand response (DR), renewable energy resources, and present a MILP for EMS. The GAMS technique is used to resolve the multi-objective optimization problem for energy management [126].

#### 4.3.2. SNOPT Solver

SNOPT and GAMS are capable of solving the nonlinear problems of the system [127]. It uses a sparse sequential quadratic programming (SQP) approach to approximate the Hessian of the Lagrangian with restricted quasi-Newton assumptions. It is particularly useful for nonlinear issues including expensive to evaluate functions and gradients. Although the functions must be smooth, they do not have to be convex. Various mathematical programming is solved by SNOPT solvers like EMS uses coordinated GAMS to ensure effective coordination and operation of the MMG system [128], SNOPT used a semidefinite QP Solver with limited memory approximation technique of Quasi-Newton and also used a reduced Hessian sub-algorithm to solve the QP sub-problems.

#### 4.3.3. Gurobi Optimizer

Mixed-integer linear programming (MILP) formulates the restoration problem of various network and TESS constraints solved using Gurobi optimizer. Gurobi optimizer is used to resolve the problems of MILP and MINLP [129,130]. The Gurobi Optimizer is commercialized for mixed-integer linear programming (MILP), quadratically constrained programming (QCP), quadratic programming (QP) [131], linear programming (LP) [132], mixed-integer quadratically constrained programming (MIQCP), and mixed-integer quadratic programming (MIQP).

### 4.4. Test System of Validation

Evaluations were conducted to test the performance of the EMS algorithm applied to the microgrid. The microgrid systems have been summarized in the area of EMS [133,134]. Evaluation of Microgrids is based on topologies. Microgrid evaluations are applied in real life and the test system [135,136]. 

Table 4 represents these test systems’ classification from various perspectives, including single and multi-microgrids, microgrid connection with the island, and grid system and distributed generation used in a microgrid. 

It also describes the microgrid with an islanded and grid-connected mode, and while mentioning the energy sources with maximum and minimum power using different types such as DG, WT, PV, FC, etc. It also described the node system for many IEEE buses.

## 5. Research Challenges

From the above analysis, it is deduced that the EMS is a very important part of campus microgrids. This paper observed different energy resources and storage systems that deal with the challenges while implementing their applications in the campus microgrids. However, some issues are briefly described to overcome the implementation issues of EMS within microgrids. Microgrid systems operate as an effective alternative approach for the power system that is connected using either grid-connected or island-connected modes. Similarly, transient stability issues, electricity rate uncertainty, grid reliability, voltage stability are some challenges that the microgrid system faces during its operation. Thus, microgrids install different RER’s (Renewable Energy Resources) to overcome the issues of high energy demand. It also focuses on making the system more responsible and sustainable. It is necessary to keep an effective framework for stable campus microgrids. It also enhances an EMS that should be designed to optimize the system’s efficiency. An enhanced time-of-use pricing structure is required to make power unit pricing efficient. 

The sustainability and techno-economic analyses of a campus microgrid were also examined. For higher education colleges (HEC), recent literature tries to reduce costs, maximize available resources, and reduce energy trading across microgrids. Innovative microgrid methods were used on many of the campuses studied to help enhance energy efficiency and reduce power dissipation and GHG emissions. Many campuses were studied, including Eindhoven, YZU (Yuan Ze University), University of Genova (Savona), Connecticut Microgrid Campus, University of Novi Sad, Clemson University, Illinois Institute of Technology (IIT) and Tsinghua campus, China. The literature also contains some of the most recent research for inventive researchers who want to convert traditional microgrids into intelligent grid systems.

The following are among the research challenges that campus microgrid faces:

1. To maximize the utilization of green sources on campus;
2. To minimize the campus microgrid’s operating and running costs as low as possible;
3. To ensure the system’s reliability and dependability;
4. To reduce the use of utility electricity by offering renewable energy resources;
5. To improve the system more stable by incorporating modern optimization techniques;
6. To improve an EMS that is meant to maximize the efficiency of the system;
7. To ensure electricity unit prices efficient, a better time-of-use pricing scheme is necessary;
8. To create an effective economic plan in order to increase the economic benefit of the advanced campus microgrid system.

The major goal of this research is to provide a quick summary of previous studies on campus microgrids that addresses both operating expenses and energy system usage in literatures. It also discusses EMSs (energy management systems), energy trading, and technologies that can sustain campus microgrids. It also promotes the advancement of intelligent campus microgrids through technology and research, taking into account socioeconomic advantages, suitable power flow solutions, and smart campus microgrid financing. It also focusses on campus distributed generation at these institutes, such as energy storage, wind, solar PV, and EV charging and discharging scenarios. These issues were based on realistic microgrid energy systems with a variety of approaches and deployment scenarios aimed at utilizing green energy, developing a smart campus, and achieving renewable energy for the campus microgrids to minimize GHG emissions.

It also looked at energy trading strategies among prosumers and customers in recent studies. Consumers can choose between using the utility or purchasing power from the grid, or operate in islanded mode. The goal is to increase renewable energy use while lowering ESS and generating costs, which have an economic impact on the prosumer. Investing in smart grids, which will transform current conventional campus microgrids into a smart microgrid, is the effective solution to these issues. Maintaining electricity supply is said to be critical for each campus microgrid, particularly during a grid interruption such as in outage situations. The entire microgrid system will be more efficient as a result of this. Improved power generation and load flow control will help consumers as well. Reduced GHG emissions and climate change, particularly through CO_2_ reduction, will be critical.

There are still many issues such as power management, sustainability, reliability, power quality, load shedding, voltage, and frequency stability that creates difficulties in implementing the microgrid in the system. Somehow, energy management of microgrids through optimal scheduling of distributed generation is an optimal solution to mitigate the microgrid aforementioned challenges using optimization techniques. This paper also observed different energy storage systems such as fuel cells, batteries, and electromagnetic storage devices. There are many issues in the batteries such as low life cycle, slow charging, low energy density issues, and complexity in maintenance. The solution tools need advancement to overcome the computational time issues and such related concerns. Furthermore, machine learning, artificial intelligence, and blockchain techniques are more suitable optimization techniques as compared to classical techniques. 

## 6. Conclusions

In this paper, a comprehensive survey of the campus microgrids, their optimal scheduling of the distributed energy resources is reviewed considering the limitations and solutions. This work analyses different energy resources with multiple solutions techniques proposed in the literature for energy management of various campus microgrids at different locations. It also investigates the optimal power output of various Distributed generators. Additionally, it briefly explains the voltage and frequency regulation of the MG system consideration of grid-connected or islanded modes. This paper also reviews the MG-EMS, which has evolved in recent frameworks, discussing different types of MG generation and multiple storage units installed at different locations. Similarly, the study briefly defines the MG-EMS objective functions and up-to-date optimization algorithms are also evaluated. The objective functions used in the literature are also reviewed, along with the system various components. These methods are chosen and explained with their optimal solutions based on their practicality, resource availability, and especially their reliability of the microgrid environment. The research challenges and their limitations are also addressed in the literature. Furthermore, a thorough study is also needed to address the recent problems and trends with the best possible methods and approaches available for the advanced campus microgrid energy management system such as blockchain, artificial intelligence, or machine learning.

## Figures and Tables

**Table 1:** A review on the energy management of many microgrids.

Ref.	Subject	Components	Optimization Techniques	Load Types	Results
[11]	Illinois Institute of Technology (IIT)	Distributed generation (DG), controllable loads, storage, Switch	High-reliability distribution system (HRDS)	Electrical appliances	Annual Operational cost reduces (140,497 $/year. to 126,644 $/year.)
[11]	Illinois Institute of Technology (IIT)	Distributed generation (DG), controllable loads, storage, Switch	High-reliability distribution system (HRDS)	Electrical appliances	Annual Operational cost reduces (140,497 $/year. to 126,644 $/year.)
[12]	University Kuala Lumpur, British Malaysian Institute	Photovoltaic (PV), battery storage system, Wind, Converter	Hybrid Optimization Model for Electrical Renewable (HOMER)	Typical load profile for a small campus	Economical evaluation of greenhouse gasses emissions
[13]	50 higher universities around the world	All renewable resources, energy storage system	All universities have different Techniques	Electrical load	Economic benefits
[14]	Nathan Campus, Griffith University, Australia	DG and ESS, battery bank, PV, WT, FC	Control and management system operation	AC DC Load, EV.	Energy management system
[15]	Nanyang Technological University (NTU), Singapore campus	PV, FC, and Natural-gas operated MT	Laboratory of Clean Energy Research (LaCER)	Buildings and transportation	Microgrid Energy Management System (MG-EMS
[16]	All Prosumers	ESS, PV, and wind generation	MILP, MICP	Domestic and Commercial Load	Saving in Electrical cost
[17]	Overview microgrid implementation in American, Asian and European countries.	Control system, Utility network, renewable sources, Diesel generator	Different techniques use	Electrical appliances	Power quality and reduce dependency
[18]	rural areas	Diesel generator, PV, Energy Storage Battery’s, metering	IBM ILOG CPLEX	Electrical appliances	Efficient
[19]	Modified Microgrid	Diesel generator, Wind, Microturbine, Energy Storage Battery’s, metering	(GAMOM), (PSO), (TLBO)	Electrical appliances	Economic benefits, less solving time
[20]	Modified microgrid with the usage of inverter	PV, Fuel cell, inverters	a multiagent system (MAS)-based	Electrical appliances	Reduce Communication
[21]	Industries	PV, Wind, Energy storage system, Diesel generator	MILP	Industrial load	Economic benefit
[22]	Islanded residential microgrid (MG)	Gas engine, Microturbine, PV, Fuel Cell, Energy Storage system	Two-stage stochastic programming	Electrical appliances	maximize the expected profit of MG and energy payments of customers.
[23]	Optimal scheduling Multi microgrid	MT, GE, Wind, PV, Energy storage, Fuel cell	MILP	Electrical load	Most reliably and economical
[24]	Multi-Microgrids	PV, Wind, ESS, DiG, FC	MILP, CPLEX 11 under GAMS	Electrical load	Minimize the operation costs and optimally schedule energy resources to fulfill the demand loads
[25]	To enhance the resilience of distribution systems (DS)	PV, Wind, ESS	MILP, Gurobi	EV, Domestic, Commercial Load	It minimizes power system cost, generation cost, and customer interruption cost
[26]	Multi-Microgrids with ESS	MT, PV, Energy Storage system	bi-level model Optimize Problem, (GAMS)	Electrical load	Reduce the operational cost and maximize the owner profits
[27]	Grid-Connected Microgrid	PV, Wind, GE, ESS, MT	MINLP, NSGA	Electrical load	It maximizes the profit and reduces the GHG emissions
[28]	Electrical Thermal resources in microgrid	GE, PV, ESS, Wind, converter, inverter	MILP	Thermal, Electrical load	It minimizes the operation costs
[29]	AC/DC Hybrid Multi-microgrids	DiG, ESS, PV, Wind	YALMIP toolbox of MATLAB and CPLEX solver 12.4	Electrical load	Economic benefit
[30]	scheduling flexible resources in microgrids operation	ESS, PV	MOSEK SOCP	Electrical load	Economic benefit

Some Pros and Cons of the Literature review components are mentioned here: Wind Power: Pros: Reliable. Cons: Expensive to be installed and the wind does not operate continuously. PV: Pros: Free energy available in nature. Cheap energy once installed. Cons: Expensive. Efficiency level low, as it requires converters and storage devices which are also expensive. Fuel Cell: Pros: Fuel cells are 85% energy efficient. Cons: Faces problems in productivity and storage of hydrogen gas. Battery energy storage system: Pros: Maintenance costs less. Simple charging algorithm. Low discharging time. Cons: Degrades at high temperature and limited cycle life. Micro-Turbine (MT): Pros: Easy installation. Easy maintenance and operations. Cons: If loaded, it can be heated early. Gas Engine (GE): Pros: It has an efficient engine design for small-scale and large-scale engines. Cons: Lower thermal efficiency.

**Table 2:** Comparison of optimization methods considering advantages and disadvantages.

Techniques	Optimization Methods	Advantages	Disadvantages	Applications and Objectives
**Deterministic Techniques**	MILP [46]	The problems are swiftly and completely resolved using mixed-integer linear programming (LP). Their linear constraint is located in the viable convex area, with the goal of locating the best global point and precise solution.	Economic and stochastic analysis are two types of analysis. It has limited capabilities for applications with objective functions that are not continuous or distinct.	For optimization challenges, MILP is often utilized. It’s simple to operate with CPLEX Solver, that is a good piece of software. Unmanned aerial vehicles (UAVs) utilize it to design their flight trajectories.
	Dynamic Programming (DP) [47]	To divide the difficulties into smaller components and then optimizing them to obtain the best answer	It is time-consuming since it has a huge number of recursive routines.	It is also employed as an issue of optimization. It handles issues like dependability design, robots control, and navigation systems, among others.
	MINLP [27]	Solve issues using basic operations and has a large number of optimum solutions that outperform MILP.	It takes a long time.	Mixed-integer nonlinear programming (MINLP) is a method for solving optimization problems containing continuous and discrete variables in the optimization problem, as well as complex variables.
**Metaheuristic** **Techniques**	Particle Swarm Optimization (PSO) [48]	Greater productivity while fixing optimization issues. Easy adaption for a variety of optimization issues and timely reporting of an optimal alternatives.	When addressing an optimal solution, complex calculation is required. In small optima/minima zones, the searching process may get entrapped.	Many optimization issues, such as power management, may be solved with PSO. It may also be utilized for video graphical effects.
	Genetic algorithms (GA) [49]	Focused on population evolutionary computation, which use mutation, selection, and crossover to find the best solution. They do also have a fast convergence rate and can rapidly adapt to different types of optimization techniques, providing near-optimal outcomes in a fair amount of time.	While resolving, the requirements for the selection, mutation, and crossover processes must be satisfied. It also does not ensure that the best solution will be found. Similarly to PSO, the search process may become entrapped in localized optima/minima areas.	In natural sciences, such as architectures, genetic algorithms can be used to find a comprehensive solution. It is employed in image processing as well as learning the robot’s behavior. It is also utilized in distributed applications for data allocation.
	Artificial Fish Swarm [50]	High precision, few variables, flexibility, and quick convergence are all advantages. It also adapts well to a variety of optimization situations, producing near-optimal approaches in a fair amount of time.	It has the same benefits as genetic algorithms, but it has drawbacks because to the lack of mutation and crossover. It is also no assurance that you will find the greatest answer. Furthermore, similarly to GA, the searching may become entrapped in specific optima/minima areas.	Fault tolerance, quick convergence speed, outstanding adaptability, and great precision are all advantages of artificial fish swarms. It frequently uses the general technique to tackle a variety of issues, including prey, followers, and swarms. Neural network learning, color quantization, and data segmentation are some of the other uses of AFS.
**Artificial Intelligence Techniques**	Artificial Neural Network [51]	Its evaluation time is quicker than prior algorithms, and it solves difficulties such as obtaining target objective functions for real-valued, binary, and other values.	It supports parallel processing and is hardware technology dependent. It provides unexpected answers but no indication of how they were achieved.	Handwriting recognition, picture compression, and stock exchange predictions all employ deep neural networks.
	Fuzzy Logic [52]	Fuzzy logic’s structure is simple to grasp, which makes it appealing to engineers who want to use it to operate machines.	It can be challenging to maintain precision while using fuzzy logic.	Fuzzy logic is widely utilized in spaceflight, the automobile industry, traffic control, and, most notably, in enhancing the transmission system’s performance.
**Special** **Techniques**	Manta Ray Optimization [53]	When compared to alternative optimizers, the computing cost is lower, and the results are more precise.	Its fine-tuning for finding solutions for optimization is ineffective, and its convergence rate is extremely slow, finding it less useful.	The manta ray approach is a bio-inspired optimizing algorithm inspired by the exceptional behavior of gigantic manta rays recognized for their rapidity. It is popular because of its high accuracy and low computational cost.
	Harris hawks Optimization [54]	It is well-known for its good performance, reasonable convergence, and high-quality optimization outputs.	It can be tough to grasp at times, and the computing complexity adds to the difficulty.	HHO is still in its early stages for academics, but it offers good convergence, precision, and speed for addressing real-world optimization issues.

**Table 3:** A review on the objective functions of various energy management systems.

Ref	Objectives Functions	Details
[97]	$COE=\frac{C_{antot}}{E_{anserved}}$	The objective function consists of COE that represents energy cost which is calculated as: total annualized cost ($C_{antot}$)/total annual energy served ($E_{anserved}$). The main problem is to calculate the energy cost and use optimization algorithms to solve it. It can also add some other costs like NPV (Net present Value) analysis.
[98]	$F=\overset{m}{\underset{t=1}{\sum }}\left(C_{t}^{g}+Cr_{t}^{g}+C_{t}^{ES-}-C_{t}^{l}-C_{t}^{ES+}+\Omega_{t}\right)\times ∆t$	It consists of $C_{t}^{g}$ that is the renewable energy cost and $Cr_{t}^{g}$ is the non-renewable energy cost. $C_{t}^{ES-}$ is the cost of ESS charge and, $,C_{t}^{ES+}$ is the discharge cost of ESS. $C_{t}^{l},\;\;$ is the DR cost and $\Omega_{t}$ is the penalty of the energy not supplied. Its problem was to calculate the renewable energy cost. It lacks some resources, like PV, wind which costs can be added, if a microgrid enhance it by incorporating more resources and in this way, cost efficiency could be increased.
[99]	$F=NPC+\overset{8760}{\underset{t=1}{\sum }}P_{b}\left(t\right)+\overset{8760}{\underset{t=1}{\sum }}P_{H_{2}}\left(t\right)+\overset{8760}{\underset{t=1}{\sum }}P_{w}\left(t\right)+P_{wt}+P_{H_{2}T}$	The main objective function relies on NPC which is the net present cost for twenty operating years. $P_{wt},P_{H_{2,}}P_{H_{2}T}$ are the battery, water, water tank, hydrogen, and metal hydride tank penalty, represent.
[100]	$F=CF_{t}^{OPR}+CF_{t}^{EMI}+CF_{t}^{RLB}$	This objective function consists of $CF_{t}^{RLB},CF_{t}^{EMI},\;\;CF_{t}^{OPR}$ represent the emission, reliability and operation cost of microgrid.
[101]	$F=C_{in}^{MG}+C_{op}^{MG}\;C_{op}^{MG}=\overset{L}{\underset{i=1}{\sum }}\left(C_{Fi}+C_{OMi}+C_{Si}+C_{Ei}\right)+\overset{M}{\underset{j=1}{\sum }}C_{OMj}^{ESS}-C_{G}^{MG}$	This EMS cost composed of $C_{in}^{MG}$ is the investment cost and $C_{op}^{MG}$ is the operation cost. However, it can also add maintenance cost to further analyze the EMS cost.
[102]	$F=Cost^{Operating}+Cost^{Emission}\;Cost^{Operating}\;Cost^{Emission}=\overset{T}{\underset{t=1}{\sum }}\left\{emission_{DG}\left(t\right)+emission_{s}\left(t\right)+emission_{Grid}\left(t\right)\right\}$	The objective function of the microgrid is considered as an emission and operating cost. More cost can be added, if the microgrid involves PV, it will also make a system towards efficiency.
[103]	$F=F_{Cost}^{start-up}+F_{Cost}^{reserve}+F_{Cost}^{generation}+F_{Cost}^{DR}+F_{Emission}$	The objective function of the microgrid is composed of emission functions and overall cost. It lacks investment cost and operational and maintenance cost, which is necessary for a system.
[104]	$Frequency_{MG}=\overset{Ns}{\underset{s=1}{\sum }}\pi_{s}\left(\overset{Nh}{\underset{h=1}{\sum }}\underset{l}{\sum }\left|\Delta f\left(s,l,h\right)\right|\right)$	It consists of $Frequency_{MG}$ that controls MG frequency as the EMS OF.
[105]	$F=\omega_{1}\overset{T}{\underset{t=1}{\sum }}\cos t^{t}+\omega_{2}\overset{T}{\underset{t=1}{\sum }}Q_{r,i}Emission^{t}$	I is the price penalty factor while $\omega_{1}$ and $\omega_{2}$ are the non-negative coefficients for adjusting objective functions.
[106]	$F=\overset{T}{\underset{t=1}{\sum }}\left\{\overset{N}{\underset{n=1}{\sum }}\left(P_{n,t}B_{n,t}+SU_{n}\times y_{n,t}+SD_{n}\times z_{n,t}+\;\;\;c\pi_{n,t}^{U}SR_{n,t}^{U}c\pi_{n,t}^{D}SR_{n,t}^{D}\right)+\overset{ND}{\underset{d=1}{\sum }}CDR_{d,t}+\overset{S}{\underset{s=1}{\sum }}Pr_{t,s}SC_{t,s}\right\}$	The cost function composed of, star-up costs, shut-down costs, and generation trade-off of DGs as well as security cost of the network and up and down reserves of demand response. However, if NPV and COE cost can be focused, it may take the system towards cost efficiency.
[107]	$F=\underset{t\epsilon T}{\sum }C_{t,money}+\underset{t\epsilon T}{\sum }C_{t,money}^{startup}-\underset{t\epsilon T}{\sum }P_{t,money}+\underset{t\epsilon T}{\sum }\underset{t\epsilon T}{\sum }\mu_{t,g}.\pi_{g}$	It consists of $C_{t,money,\;}$ is the operation cost and $C_{t,money}^{Startup}$ represent the start-up costs while $P_{t,money\;}$ denote the total revenue. Last term denotes the penalty of the unmet load. Lastly, investment cost must be focused in a system which is also a necessary component.

**Table 4:** Survey on different IEEE Microgrid test systems.

Ref	Microgrid Mode		Energy Source			Node System
	Islanded	Grid-Connected	Type	Min Power	Max Power	
[99]	✘	✔	MT	0 MW	0.8 MW	IEEE 33
			PV	0	275 kW	
[100]	✔	✔	WT.	200 kW	300 kW	IEEE 34- node systems
			PV	80 kW	120 kW	
			ESS	−20 kW	200 kW	
[137]	✔	✔	DiG	100 kW	790 kW	IEEE 33 bus system
			WT	8000 kW	45,000 kW	
[138]	✔	✔	DiG	1.60 MW	1.80 MW	IEEE 33 bus system
			BES	0	0.2 MW	
[139]	✔	✔	PV	0	11 MW	IEEE 84 bus system
			MT	0	5 MW	
			ESS	0	8 MW	
[140]	✘	✔	DiG	0.5 MW	5 MW	IEEE 33 bus system
			MT	0.1 MW	2 MW	
[141]	✘	✔	BES	11.93 kW	19.40 MW	IEEE 6 bus system
			DG	200 kW	300 kW	
[142]	✘	✔	MT.	0 kW	1000 kW	IEEE 33 bus system
			WT	0 kW	1000 kW	
			PV.	0 kW	1500 kW	
			ESS	−1500 kW	1500 kW	
[143]	✘	✔	─	─	─	IEEE 30 bus system
[144]	✘	✔	PV	16.2 kW	77.6 kW	IEEE 33-bus distribution network
[145]	✘	✔	DiG	10 kW	100 kW	IEEE 33-bus test system
			ESS	0 kW	16.6 kW	
			EV	0 kW	111 kW	
			PV	0 kW	126.8 kW	
[146]	✘	✔	─	─	─	IEEE 33

## Data Availability

Not applicable.

## Nomenclature

The following acronyms and nomenclature are used in this manuscript:

**Acronyms**	
EMS	Energy management system
ESS	Energy storage system
DOD	Depth of discharge
FIT	Feed-in-Tariffs
BESS	Battery energy storage system
BSOC	Battery state of charge
DG	Distributed generator
DERs	Distributed energy resources
DSM	Demand-side management
GHG	Greenhouse gas
LP	Linear programming
PV	Photovoltaic
MILP	Mixed integer linear programming
TOU	Time-of-Use
RERs	Renewable energy resources
μG	Microgrid
FLC	Fuzzy logic controller
SOC	State of charge
